# Preparation and Characterization of Char Carbon Obtained by Carbonization of Unused Cigarette Filter Rods: The Product Application Assessment

**DOI:** 10.3390/ma18071661

**Published:** 2025-04-04

**Authors:** Bojan Janković, Dejan Cvetinović, Milena Milošević, Filip Veljković, Vladimir Rajić, Marija Janković, Vladimir Dodevski

**Affiliations:** 1 University of Belgrade, “Vinča” Institute of Nuclear Sciences, National Institute of the Republic of Serbia, Mike Petrovića Alasa 12-14, P.O. Box 522, 11001 Belgrade, Serbia; bojan.jankovic@vinca.rs (B.J.); deki@vinca.rs (D.C.); filipveljkovic@vinca.rs (F.V.); vladimir.rajic@vinca.rs (V.R.); marijam@vinca.rs (M.J.); 2 University of Belgrade, Institute of Chemistry, Technology and Metallurgy, National Institute of the Republic of Serbia, Njegoševa 12, 11000 Belgrade, Serbia; milena.milosevic@ihtm.bg.ac.rs

**Keywords:** cigarette filter rods, carbonization, char, high carbon yield, oxygen-containing functional groups, n-alkanes, micro-/mesoporous material

## Abstract

The development of carbonaceous materials such as biochar has triggered a hot spot in materials application. In this study, a new type of char carbon was developed from raw cigarette filter rods (CFRs) via a carbonization process under moderate conditions (*T* = 550 °C; *t_res_* = 1 h) (CFR char carbon). The produced char was characterized by ATR-FTIR (Attenuated total reflectance—Fourier-transform infrared) spectroscopy, XRD (X-ray diffraction) analysis, GC-MS (Gas Chromatography–Mass Spectrometry), FESEM-EDS (Field-Emission Scanning Electron Microscopy—Energy-dispersive X-ray spectroscopy) technique, XPS (X-ray photoelectron spectroscopy), and N_2_ adsorption/desorption (BET) measurements. The obtained carbon material is rich in oxygen-containing functional groups (i.e., C=O, C–O, –C(=O)–CH_3_, C–O–C, C–OH, and O=C–O, with chemisorbed oxygen), containing significant amounts of calcium (that originates from CaCO_3_) and silicon (Si), generated by reduction of SiO_2_. It was found that the formation of char(C)/n-alkane composite material makes that CFR char have a high compressive strength improvement. Moderate carbonization has contributed to the creation of such material that has a fairly high specific surface area (320.93 m^2^/g), exhibiting a complex hierarchical structure that was characterized by composite Type I/IV(a) isotherm, associated with micro-/mesoporous carbon material. In addition, more directional extensions of this research for future work were proposed, including the implementation of electrochemical research.

## 1. Introduction

Cellulose acetate (CA) filter was added to cigarettes in the 1950s in the wake of increasingly convincing scientific evidence that cigarettes caused lung cancer and other serious diseases.

The problem related to CA in filters is that they are not biodegradable [1]. In addition to CA, commercial filter cigarettes contain wrapping paper, plasticizers, and sometimes activated charcoal. So, looking at this in general, around 98% of the materials used in cigarette filters have poor biodegradability. Considering the anatomy of the cigarette filter, Harris’ work [2] describes the development and construction of the CA filter, where CA fibers are produced by treating raw cellulose, usually obtained from the wood pulp, with acetic anhydride (a common acid reagent) in the presence of a catalyst. Details in production ultimately point to the formation of multiple solids, the uniform strands of CA filaments. Considering unused cigarette filters as well as used ones (known as cigarette butts (CBs)) and including their main component, they all fall into the category of biomass waste. Without referring to the toxicity that filters can cause after use (during the combustion of tobacco in cigarettes), from both environmental and public health standpoints, which is generally known [3], at this moment, the key question here is: How to efficiently recycle unused and used filters, and obtain new products that can be used for high-quality purposes, especially in their sustainable valorization? Today, in most cases, the sustainable valorization of CB waste is achieved through the pyrolysis process [4]. This utilization is primarily correlated with environmental impact, especially for making carbon-based adsorbents of different performances [5,6,7]. In addition, the carbonaceous by-product (biochar) arising from the thermo-chemical conversion of used cigarette filters can also be exploited in direct carbon solid oxide fuel cells [8], as well as for porous activated carbon materials for supercapacitors [9], the pyrolysis conversion of the cigarette butts also leads to the generation of ester-rich bio-oils [10].

It should be noted that the largest number of scientific papers is related to the valorization of CBs that yield valuable products, converting them via the pyrolysis process. The field of application of the resulting products is, therefore, closely related to the structure of CBs. The researchers’ efforts to recycle CBs were influenced by the physical structure and chemical properties of CBs.

The carbonization process is primarily concerned with obtaining solid char carbon as a primary product. However, both of them (pyrolysis and carbonization) are carried out in the same temperature range, although residence times and heating rates might vary. So, the main and only difference lies in the objective of the process: the carbonization goal is to maximize the char solid product, while the pyrolysis aims to maximize liquid hydrocarbon production. Consequently, CA-derived initial raw materials are good candidates for carbon materials synthesis through pyrolysis carbonization [11], providing a possible way for their recycling. In addition, carbon materials are commonly employed for the fabrication of efficient supercapacitor electrodes, and green procedures are preferred for this purpose. There are a large number of practical cases regarding this application, which can also be seen in good review works today [12,13].

Through a literature search, to the author’s knowledge, there are no data related to the thermal conversion via the carbonization of unused cigarette filters (in the form of cylindrical pellets) in the production of carbon materials. Namely, as precursor materials are used, the CA filter (white) rods (FRs), whose vital purpose is the retention of typical smoke components. These filter rods, on the commercial side in the packaging, are not expensive and already belong to low-cost precursors, so there is an economically viable solution for the development of methods based on their physical or chemical recycling. Therefore, a new type of carbon material can be produced by the application of FR recycling in thermal activation through the carbonization process, which will undoubtedly create a value-added product.

In this work, we have made efforts to present a procedure for the synthesis of char carbon by implementing a medium-temperature one-step carbonization process, as well as a full physicochemical characterization of the obtained product. The average temperature was chosen as the initial operating temperature to preliminarily determine its effect on the physicochemical properties of the obtained char carbon (in this paper, high temperatures were not applied, given the greater comprehensiveness of all tests, and considering that this is the first work on the development of novel designed carbon material, with this type of feedstock). In addition, the aim of this research is to compare the textural and morphological characteristics as well as chemical structure of the obtained carbon material from FRs as raw precursor feedstock with that carbon material obtained by using CBs as starting feedstock [14], which were produced by carbonization process, under different experimental conditions (the same residence time, but different values of operating temperatures and heating rates). The following analytical instrumental techniques were used for carbon characterization in this work such as ATR-FTIR (Attenuated total reflectance—Fourier-transform infrared) spectroscopy, X-ray diffraction (XRD), Gas Chromatography–Mass Spectrometry (GC-MS), SEM-EDS technique (the combined technique that uses a scanning electron microscope and energy-dispersive X-ray spectroscopy to analyze the obtained char carbon), X-ray Photoelectron Spectroscopy (XPS), and BET (Brunauer–Emmett–Teller) analysis (used to determine the specific surface area of solid material, based on gas adsorption measurements). Finally, based on the obtained results, an assessment of the applicability of the produced carbon material was performed. An analysis was carried out directly, straightforwardly, without “blessing in disguise”, i.e., through the route: end result → the current performance (within the given experimental conditions) → possible application. This study would mark a pioneering work that gives other authors an opportunity to improve procedures further, both in preparation and further modifications of carbon material produced by the carbonization of unused cigarette filter rods (CFRs).

## 2. Materials and Methods

### 2.1. Precursor (Feeding) Material

**Figure 1:**
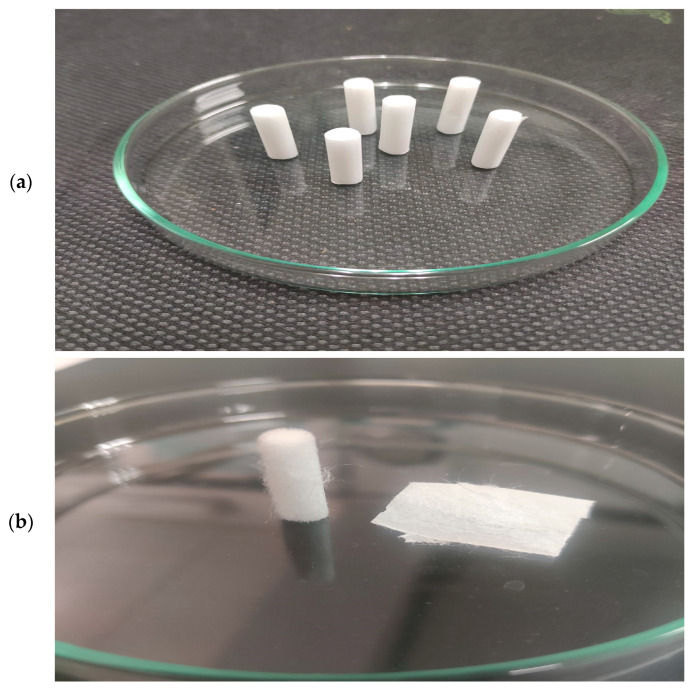
(**a**) Raw white CFRs (set of experimental samples); (**b**) The single experimental specimen: divided into three main components, such as CA fibers bundle, which is mechanically packed into a cylinder (left), the plug wrap paper (porous, which is used as filtration material (i.e., filter tow and plasticizer), which wraps the outer layer of the cigarette filter plug), and the tipping paper that holds the filter to the cigarette rod (right: plug wrap paper—edge parts, and tipping paper—central part).

Unused white cigarette filter rods (“Smoking Slim Long Paper Filters 120”—filters pack of 120 ultra-thin CA cigarette filter rods, 22 mm long and 6 mm wide; SD International, Country origin: Spain, EU) were used as raw material for their recycling into charred carbon. The advantage of these filters compared with CBs is that they are deprived of any strong leaching process, which represents a serious challenge. Namely, there is no leakage of toxic chemical compounds trapped in cigarette filters after use. Therefore, this problem is avoided here so that these compounds do not influence the structure formation of the output carbon material after the thermal process. So, there are no challenges related to recycling of hazardous waste, such as CBs. In this sense, as part of a certain pre-treatment of the samples, no additional mechanical removal of tobacco residues and/or ash was applied, nor was any rinsing to remove pollutants from ‘smoked’ filters. The appearance of the raw experimental samples (CFRs) is shown in Figure 1a.

Acetate filter rods (FRs) (Figure 1a) can effectively trap harmful substances in the smoke, so its rational design of an effective FR is important to reduce the concentrations of harmful components in the smoke. The raw experimental sample represents the cellulose acetate (CA) fibers (Figure 1b, left) derived from wood pulp. Filters have a high-density fiber structure that effectively traps the particles and gases. In addition to CA fibers, the whole filter contains filter papers, classified as plug wrap paper and tipping paper, respectively (Figure 1b, right). In this case, the cigarette paper is excluded. Highly porous plug wrap paper serves in the production of a filter-ventilated system. This paper is used to wrap the cigarette plug in order to provide ventilation while tipping paper is printable and glueable; the whole FR includes dense CA fibers with a porous plug wrap paper and perforated tipping paper (Figure 1b). It should be noted here that no component was separated, so in the pre-treatment of these samples for carbonization, the complete FRs were included. It should be noted that this study did not characterize the raw material since its composition was examined in our previous study, which is related to the upcycling of the used CB filters through the pyrolysis process [14]. This research is strictly focused on the characterization of char carbon derived from FRs (Figure 1). The current study is focused on elucidating and observing differences in the structure and physicochemical properties of the carbon material fabricated herein from unused CFRs that were obtained from the CBs examined in our previous study [14].

### 2.2. Preparation of Raw Material for Experimental Testing and Feedstock Characterization by the Proximate and Ultimate Analysis

The unused cigarette filters from two packs containing 120 rods each are prepared so that they are ground using a mechanical shredder and set up in order to prepare particles up to 12 mm. The visual inspection of the entire sample showed that it consists of typical CA fibers, with traces of the plug wrap paper and tipping paper. Such a sample was further directly used in the carbonization process. For the purpose of examination of certain properties of the raw (feeding) material (prior to carbonization), it is necessary to obtain information that will help decide whether the initial material (carbon precursor) is a decent candidate for the production of char carbon. This type of testing includes proximate and ultimate analysis.

For the experimental laboratory tests, the samples were considered as the received (*ar*.), as determined, and on a dry basis (*db*.). Proximate and ultimate analyses were performed in accordance with ASTM D7582-15, ASTM D5373-21, and ASTM D3176-15 standards. An elemental analyzer CHN628 LECO (LECO Corporation, 3000 Lakeview Avenue, St. Joseph, MI, USA) was used for the determination of carbon, hydrogen, and nitrogen contents in the ultimate analysis, which helps to characterize the investigated material and provide information about its utilization. The high-temperature combustion technique allows for sulfur (S) analysis times of less than two minutes. Table 1 shows the results of the proximate and ultimate analyses of the raw CFRs (Figure 1a).

According to the proximate analysis, the initial (precursor) material has a high content of volatile matter (VM). This can facilitate the development of the porosity within the char carbon. The CFRs have a very small content of ash (Table 1). This represents an advantage for the carbon precursor. These two features of CFRs are favorable properties for the preparation of char carbon. On the other hand, a fixed carbon (FC) content belongs to a lower value, but with regard to the type of raw material, it is still satisfactory.

The CFRs have high contents of carbon (C) and oxygen (O) but low content of hydrogen (H) (Table 1). The calculated values of H/C and O/C ratios of CFRs were 1.398 and 0.675, respectively, which fall within the typical range of biomass waste feedstock. The ultimate and proximate analyses conducted indicate that the CFRs should represent an ideal feedstock for its thermo-chemical conversion into a high-quality carbon material via the carbonization process (for the efficient use of biomass waste in a thermal process, a higher H/C ratio is more desirable than the O/C ratio).

### 2.3. Carbonization Process

Carbonization is the art of reinventing the waste material into a carbon/energy-rich charcoal. Char can be produced by a thermal decomposition process in which the feedstock is heated in an inert atmosphere to high temperatures until absorbed volatiles are expelled, thus enriching its heating value and energy content. Carbonization itself is an old process that has been used till now, but the renewed interest in it, especially with biomass waste, is because it opens new doors for commercial and scientific applications. Carbon can be “extracted” from the produced char to form the precious materials graphite and/or graphene. Namely, interpreting in general, the slow pyrolysis processes include the carbonization process. Carbonization processes can vary, both in terms of methodology and process parameters. The main objective of the carbonization process is to obtain the maximum attainable fixed carbon, but this comes on account of the product, which will suffer low energy yield, energy density, and hydrocarbon content (as opposed to, for example, torrefaction). So, based on the product motivation of carbonization and torrefaction processes, they differ in their process parameter settings in terms of temperature range and residence time, which produce carbonized and torrefied final products, respectively. Carbonization operating temperature and time are much higher than torrefaction. In the case of carbonization, this process removes almost all volatiles in the feedstock. In most cases, the carbonization process occurs slowly, heating the feedstock to high temperatures exceeding *T* = 400 °C and for several hours. The products from this process can be divided into four governed categories: (a) charcoal, when it is used as fuel; (b) biochar, when used as fertilizer or for soil amendments; (c) bio-coke for metal extraction; (d) activated carbon when the regular charcoal is upgraded for adsorption and purification purposes.

This study used a moderate carbonization process, where the operating temperature does not exceed 600 °C, where the high-temperature carbonization can be considered as one that exceeds a temperature of 600 °C. It should be noted that this classification, which includes low-, medium (moderate), and high-temperature carbonization processes, is variable, depending on the type of raw material, the type of carbonization furnace used, the heating programs used, the amount of experimental raw material fed into the furnace (carbonization furnace capacity), and other factors.

The main motive for constructing the experimental designs in this work is to determine to what extent lowering the operating temperature and reducing the heating rate affects the structure and, therefore, the type of carbon material obtained, compared with that produced under different conditions in the case of CB waste [14]. Namely, the higher carbonization temperatures resulted in larger pores and an increased aromatic/aliphatic carbon ratio in char. One of the possible pathways will be lowering the carbonization temperature to create an elevated contribution of micropores from the amorphous carbon structures. It must be clearly pointed out here that in addition to the effect of temperature, the effects resulting from the type of feedstock have a supreme role since the char carbon produced at lower and/or medium temperatures can display a more diversified organic character because of the occurrence of aliphatic and cellulose-type structures.

In this study, the carbonization equipment (the process reactor’) was used to provide lower installation costs, higher efficiency, and reliability, enabling an ideal solution for waste utilization and recycling. Carbonization is suitable for energy saving, i.e., the carbonization process produces a positive energy balance, and the entire self-heating process can be sustained only by the energy from FRs. So, the selected process allows the formation of high-value derivative(s), apropos, in this case, the high-value char, but of course, this depends a lot on the feeding raw material quality. The following advantage of the chosen procedure makes the carbon dioxide (CO_2_) balance. In other words, the carbonization process converts most of the elemental carbon into a long-term stable solid form, thereby reducing the emission of CO_2_ into the atmosphere as a greenhouse gas.

The carbonization process was carried out in a horizontal stainless steel fixed-bed reactor (Protherm Furnaces, model PTF 16/38/250, Ankara, Turkey) (tube furnace used for laboratory experimentation). In the ceramic cup, which is suitable for working with organic substances and their conversion into carbon or carbon-containing residues, around 100 g of the initial feeding material was placed. This pattern carrier was then entered inside the reactor, whereby the heating was performed in an oxygen-free environment. During carbonization, the products of organic polymer material burn out and are vented from the furnace. Since the carbon reacts with air at pyrolysis temperature(s), nitrogen or argon gas atmospheres are required in the carbonization furnace, often not allowing more than 100 ppm of the oxygen in the furnace chamber, which requires tight sealing of the furnace chamber. Therefore, the carbonization was performed in purified nitrogen (N_2_) at a gas flow rate of *ϕ* = 200 cm^3^/min, and N_2_ was used as the purge gas. The space inside the furnace was flushed before heating with dry nitrogen for 45 min., in order to remove any traces of oxygen. During the programmed linear heating mode, the reactor temperature was raised up to *T* = 550 °C, which represents the working operating temperature. When the operating temperature reached the desired value, this temperature value was maintained exactly *t_res_* = 1 h (the residence time = 1 h). The heating rate was constant throughout the process, and its value was *β* = 3 °C/min. Upon completion of the carbonization process (when char carbon was extracted from the reactor), the gaseous flow of N_2_ in the reactor was maintained during cooling until reaching room temperature. Namely, the heating rate of the feedstock has a strong influence on the property of the char carbon produced. A slower heating rate gives a higher yield of synthesized carbon and encourages the formation of aromatic structures, which renders more stability to the char. The applied slow heating rate (~3 °C/min) favored the solid residue with an increased higher heating value (HHV) and lower O/C and H/C ratio [15], compared with the fast heating rate (e.g., 20 °C/min). A slow heating rate can provide enough reaction time to promote the decomposition of biomolecules and the recombination of the intermediates. The combination of the heating rate and final operating temperature has a strong impact on the quality of the ultimate carbon material. Considering the applied experimental design, after carbonization, the yield of produced char carbon was *Yield*_(char-carbon)_ = 17.9–18.6%. The char carbon yield (as a percentage) was calculated according to the formula presented by Altıkat et al. [16]. The obtained char carbon yield (%) in our experiment was higher than the usual yield of the char derived from biomass, which is small and amounts to approximately 12% of total feedstock [17].

**Figure 2:**
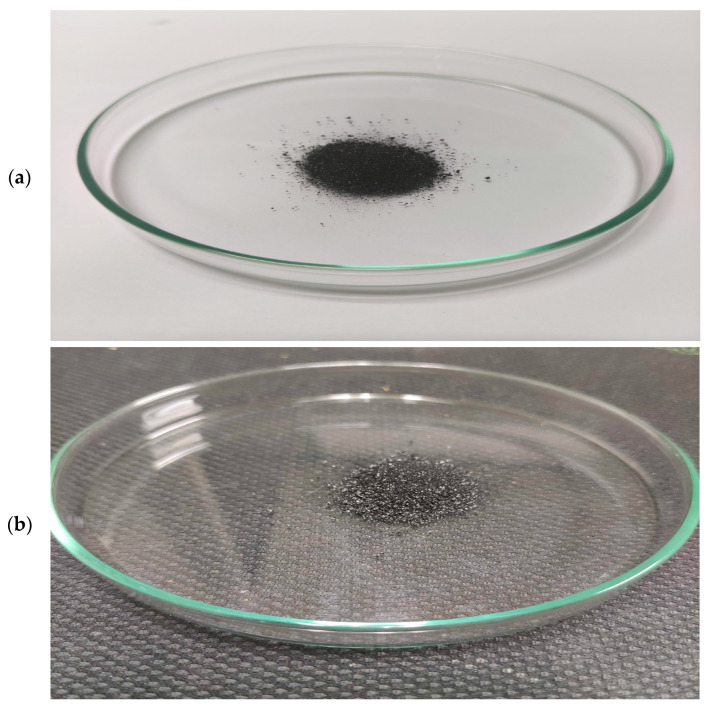
The external appearance of the manufactured char carbon from CFRs, through the carbonization process at *T* = 550 °C and *t_res_* = 1 h (sample code: ‘CFR char carbon’): (**a**) the pattern on a white background, and (**b**) the pattern on a black background.

The appearance of synthesized char carbon by the one-step carbonization using CFRs is shown in Figure 2. The produced carbon specimen is displayed against white (Figure 2a) and black (Figure 2b) backgrounds to provide noticeable contrast.

It should be noted that the produced char carbon sample contains tiny white spot particles originating from an essentially incomplete degradation process influenced by thermal performance. Namely, the latter probably belongs to the presence of metal impurities as inorganic phases in the form of oxides and/or salts, which at a given temperature can significantly influence the rate or mechanism of cellulose product degradation. An important property of cellulose products, such as bio(char) as a product of great commercial importance, is deterioration due to aging and degradation effects. Therefore, the degradation paths of cellulose and its derivatives are not the same at temperatures below and above 200 °C, especially if there are metal traces that can play the role of a catalyst during temperature intensification. Similar situations apply when organic material decomposes at temperatures below and above 600 °C, which largely depends on the activation of metals present at given temperatures as catalysts with moderate and/or strong activity. As the temperature increases beyond 400/450 °C, we can expect a substantial loss of hydrogen nuclei during the degradation of cellulose-based material, which may be reflected by a decrease in the H/C ratio, especially at a temperature of 500 °C with longer residence times. On the other hand, this also leads to an increase in molecular mobility, primarily from the anti-plasticization of cellulose.

### 2.4. Instrumental Techniques for the Physicochemical Characterization of Synthesized Char Carbon from CFRs

To improve the overall performance and fully realize the possible applications of carbonaceous material produced from the carbonization process of CFRs, accurate information about the microstructure and chemical behavior must be known. Material characterization provides information regarding the microstructure and functional groups characteristic of a particular product, thus confirming the formation and reaction mechanisms that occur, where the latter can help in its classification for practical applications. The structural and chemical nature of synthesized carbon material can be elucidated via the characterization using various analytical methods. This study includes characterization through methods such as ATR-FTIR, XRD, GC-MS, SEM-EDS, XPS, and BET analysis. Thus, this study provides the first information about the textural, morphology, and chemical characteristics of char carbon manufactured from cigarette filter rods—CFRs. Hereinafter, the designation for the carbonized sample will be “CFR char carbon”.

#### 2.4.1. FTIR Analysis

Fourier-transform infrared (FTIR) spectroscopy was applied to the powdered carbonized sample, where the spectrum was recorded in the transmittance mode with the Nicolet^TM^ iS^TM^ 10 FT-IR spectrometer (Thermo Fisher SCIENTIFIC, Waltham, MA, USA), equipped with Smart iTR^TM^ Attenuated Total Reflectance (ATR) sampling technique (Thermo Fisher SCIENTIFIC, Waltham, MA, USA). The recording was performed in the wavenumber range of 500 cm^−1^–4000 cm^−1^, at a 4 cm^−1^ resolution, and in 20 s scan mode ramping.

#### 2.4.2. XRD Analysis

The carbonized sample (in powdered form) was characterized at room temperature by the X-ray powder diffraction (XRPD) using an Ultima IV Rigaku diffractometer (Rigaku Corporation, Scientific Instruments, Tokyo, Japan), equipped with CuKα 1,2 radiations, a generator voltage of 40.0 kV, and a generator current of 40.0 mA. The range of 5°–60° 2*θ* was used for the investigated sample in a continuous scan mode, with a scanning step size of 0.02° and a scan rate of 10° per minute, using the D/TeX Ultra-High-Speed detector (Rigaku Corporation, Scientific Instruments, Tokyo, Japan). The software package Powder Cell [18] was used to identify the phases present in the examined sample.

#### 2.4.3. GC-MS Analysis

A mass of 30 mg was measured from the carbonized sample in three head-space bottles and then dissolved in 5 mL of dichloromethane. The closed vials were subjected to extraction on the ultrasonic bath for 30 min., and then from the sample, 4 mL of the total volume was filtered, transferred to a vial, and concentrated to 0.1 mL using a gentle stream of nitrogen at room temperature. The analysis was performed using a gas chromatograph (Agilent Technologies Inc., Model 7890B, Agilent Technologies, Inc., Santa Clara, CA, USA) with a mass detector (Agilent Technologies Inc., Model 5977B, Agilent Technologies, Inc., Santa Clara, CA, USA). The GC column was an HP 5-MS (30 mm × 0.25 mm i.d., the film thickness was 0.25 μm). Helium was used as the carrier gas at a rate of 1.5 mL/min. The sample injector temperature was set at *T* = 250 °C, the solvent delay was 2.5 min., and the sample was injected at a volume of 1 µL in splitless mode. The GC oven temperature was programmed as follows: initially held at 40 °C, increased to 210 °C at a heating rate of 5 °C/min, held for 3 min., and then raised from 210 °C to 305 °C at a heating rate of 30 °C/min, and finally held at 305 °C for 5 min. The injector and detector temperatures were maintained at 220 °C and 230 °C, respectively. The mass spectrometer was operated in the full-scan electron-impact (EI) mode at 70 eV (almost all commercial GC-MS instruments equip this mode as the standard ionization). GC-MS data were analyzed using the Agilent GC-MS Mass Hunter software (https://www.agilent.com/en/promotions/masshunter-mass-spec, accessed on: 5 January 2020) and the NIST 14 mass spectral library (Agilent Technologies, Inc., Santa Clara, CA, USA).

#### 2.4.4. SEM-EDS Analyses

For investigation of the surface topography and morphology of synthesized carbon material in a powder form, the ultra-high-resolution Field-Emission Scanning Electron Microscopy (FESEM) was used (The Scios 2 DualBeam System that operates at a voltage of 30 kV, Thermo Fisher SCIENTIFIC, Waltham, MA, USA). The chemical composition of the sample was examined by energy-dispersive X-ray spectroscopy (EDS). The Scios 2 DualBeam (which delivers the best-in-class performance in sample preparation) and Thermo Scientific Auto Slice software (Thermo Fisher SCIENTIFIC, Waltham, MA, USA) allows for high-quality, fully automated acquisition of multi-modal datasets, such as energy-dispersive spectroscopy (EDS) for the compositional information, including the backscattered electron imaging (BSEI), for the maximum materials contrast.

#### 2.4.5. XPS Analysis

X-ray photoelectron spectroscopy (XPS) measurement was performed on SPECS Systems with XP50M X-ray source for Focus 500 (Spec Sys. Inc., 9 Kindon Rd, Johannesburg South, South Africa) and PHOIBOS100 energy analyzer (SPECS Surface Nano Analysis GmbH, Voltastrasse 5, Berlin, Germany) using a monochromatized Al Kα X-ray source (1486.74 eV), operating at 12.5 kV and 250 W. During the measurement, the base pressure in the system was below 5 × 10^9^ mbar. For the analysis, the sample was fixed onto an adhesive copper foil to provide a strong mechanical attachment and good electrical contact. The measurement was performed in the fixed analyzer transmission mode (FAT). The survey scan spectrum was recorded over a binding energy range from −8 to 1200 eV, with an energy step width of 0.5 eV, a dwell time of 0.2 s, and a constant pass energy of 40 eV. Detailed XPS spectrum was acquired at the pass energy of 20 eV, the energy step of 0.1 eV, and a dwell time of 2 s. The derived spectrum was collected by the Specs Lab data analysis software (https://www.specs-group.com/specs/products/detail/prodigy/, accessed on 7 April 2020), and was analyzed using the Casa XPS software package (http://www.casaxps.com/berlin/, accessed on 18 May 2020).

#### 2.4.6. BET Analysis

The textural properties of CFR char carbon were determined using N_2_ adsorption–desorption isotherm tests on a surfer gas adsorption porosimeter (Thermo Scientific Surfer Gas Adsorption Porosimeter, ThermoFisher SCIENTIFIC, Waltham, MA, USA), according to the Brunauer–Emmett–Teller (BET) theory [14]. Before passing the investigated sample into a sorption device, the carbonized specimen was degassed at the stations provided for the current operation. Brunauer–Emmett–Teller (BET) technique was used to calculate specific surface area, while Barrett–Joyner–Halenda (BJH), Cranston–Inkley (CI), and Dollimore-Heal (DH) methods [19,20,21] were used to determine the volume and diameter of mesopores in the char carbon, and finally, *t*-Plot and Horvath–Kawazoe (HK) methods [22,23] were used for micropore size analysis.

## 3. Results and Discussion

### 3.1. FTIR Results of CFR Char Carbon

**Figure 3:**
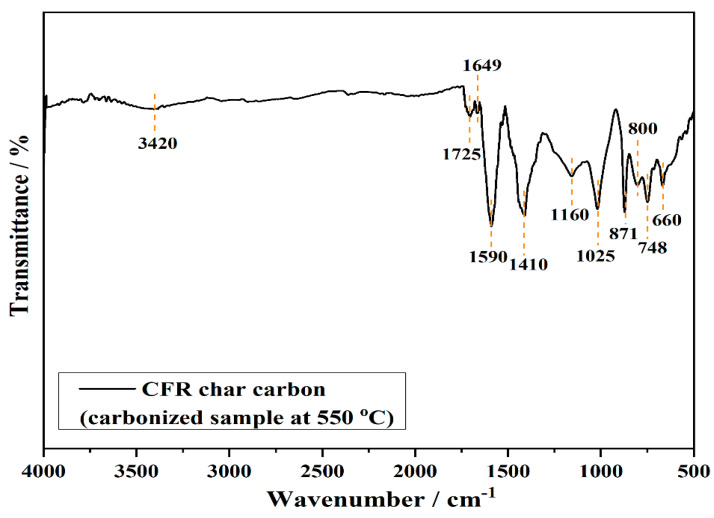
FTIR spectrum of CFR char carbon (T = 550 °C; *t_res_* = 1 h). On the same spectrum, the main vibrational bands were marked.

The FTIR technique is used for the analysis and determination of surface functional groups of the carbonized sample at 550 °C (CFR char carbon), as presented in Figure 3.

The spectrum shows characteristic FTIR signals of residual groups present in thermally treated cellulose-based materials at an operating temperature of 550 °C. The two small intensity peaks, at 3420 cm^−^^1^ and 1649 cm^−^^1^, correspond to the stretching and bending vibrations of the cellulose hydroxyl groups, respectively. In addition, the small vibrational band at 1725 cm^−^^1^ is attributed to the C=O stretching vibrations of ester groups, from CA as the main structure in CFRs [14], while the peak that appears at 1590 cm^−^^1^ represents the skeletal C=C stretching vibrations of the aromatic ring. The three vibrational bands observed at 1410 cm^−^^1^, 871 cm^−^^1^, and 748 cm^−^^1^ can be attributed to C–O stretching, out-of-plane, and in-plane vibrations, respectively, of the carbonate anion (CO_3_^2−^) [24]. These bands are characteristics of the calcite (CaCO_3_), natural limestone which, together with natural and/or synthetic products, such as titanium dioxide (TiO_2_), the aluminum hydroxide (Al(OH)_3_), and others, are used as the fillers in the paper, for the improvement of stability, appearance and mechanical characteristics [25,26]. Regarding this, the small vibrational peak in CFR char carbon FTIR spectrum, at about 660 cm^−^^1^ (Figure 3), can be assigned to Ti–O bonds in TiO_2_ [27]. Namely, it should be noted that TiO_2_ is applied to cigarette filter material as a whitening agent. TiO_2_ is bound to cigarette filter material in a manner that the TiO_2_ should not be mechanically released. Any thermal release of TiO_2_ from the filter into the mainstream smoke during cigarette consumption is prevented by the high temperatures at which TiO_2_ evaporates (roughly ~3000 °C) [28]. In addition to titanium dioxide, cigarette filters contain minerals such as CaCO_3_, such as coating color pigment and filler, especially for the enhancement of the degree of whiteness of paper [29]. Precipitated calcium carbonate is required for special papers, such as cigarette filter paper [29,30,31,32]. Among these bands, the vibrational peak at 1410 cm^−^^1^ is also attributed to the C–H bending vibrations of methylene groups from cellulose, while the bands observed at lower wavenumbers, i.e., at 871 cm^−^^1^ up to 500 cm^−^^1^, are attributed to the deformation vibrations of C–H bonds present in aromatic rings. Additionally, the peaks at 1160 cm^−^^1^–1025 cm^−^^1^ arise from acetyl ester and C–O, C–O–C from ether groups [14]. In general, the absence of bands about 2900 cm^−^^1^–2800 cm^−^^1^ for methylene groups and overlapped bands from charred cellulose structure, with inorganic components in CFR char spectrum, indicate a significant increase in the condensed aromatic structure, and the loss of aliphatic functional groups due to decomposition and dehydrogenation bonds in cellulose acetate during carbonization.

### 3.2. XRD Results of CFR Char Carbon

**Figure 4:**
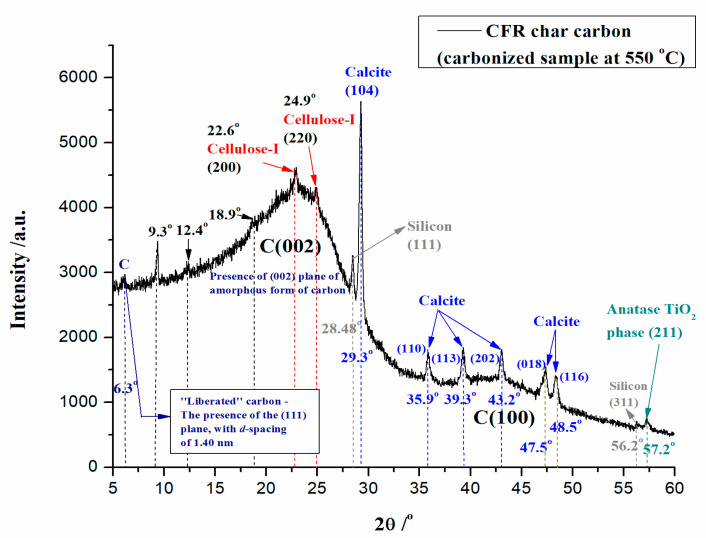
XRD pattern of CFR char carbon (*T* = 550 °C; *t_res_* = 1 h).

Figure 4 shows the XRD result of the formed carbon material by the carbonization process of CFRs at *T* = 550 °C and *t_res_* = 1 h (CFR char carbon).

From the recorded XRD spectrum of CFR char carbon, the existence of porous carbon that displays two broad peaks, around 2*θ* ~ 22° (C(002) plane) and 2*θ* ~ 43° (C(100) plane), can be clearly seen (Figure 4). This is the characteristic of the amorphous graphitic carbon. Two indicated peaks belong to (002) and (100) diffraction patterns of carbon, respectively. Considering these results, they suggest that the graphitization of porous carbon is quite weak [33]. Additionally, it can be observed that the XRD spectrum contains peaks located at 2*θ* = 22.6° and 2*θ* = 24.9° (Figure 4), which can be attributed to the reflections from (200) and (220) planes of cellulose-I, respectively [34,35,36]. Generally, the peaks at 2*θ* = 20°–30° refer to the stacking structure of aromatic layers (graphite—002), and the broadening has originated from small dimensions of the crystallites perpendicular to the aromatic layers. However, it is also indicated by the existence of sharp peaks between 2*θ* = 27.5° and 30°, which indicate that CFR char carbon contains miscellaneous inorganic components (Figure 4). Among them, one sharp peak with a stronger intensity and another peak with a weaker intensity, located at 2*θ* = 29.3° and 2*θ* = 28.48°, are attributed to calcite (CaCO_3_) (104) crystal plane [37,38]. Moreover, the elementary silicon (Si) with (111) crystal plane [39]. In addition to the observed sharp peak, the presence of calcite was noted by the appearance of peaks related to CaCO_3_ at the following Bragg’s angles: 2*θ* = 35.9°, 39.3°, 43.2°, 47.5°, and 48.5° (Figure 4), which correspond to the (110), (113), (202), (018), and (116) calcite (CaCO_3_) crystal planes, respectively [40,41,42]. Furthermore, another additional peak attributed to silicon (Si) with (311) crystal plane at 2*θ* = 56.2° [43] was noticed (Figure 4). It should be noted here that the occurrence of elemental silicon in CFR char carbon actually represents a metallic “impurity”, and it was attached to the growth of silicon crystals, which is strictly related to the transport of impurities [44]. However, the produced char carbon also helps to generate silicon, which was obtained by reducing silicon dioxide (SiO_2_) (where the reducing agent represents the char) through the following equation: SiO_2_ + C → Si + CO (single displacement (substitution) reaction). So, the carbon in the char becomes an essential reactant due to the high temperature, helping to reduce silicon to its pure form. From the presented results, it can be seen that the obtained carbonized sample has an important advantage, which includes the fact that it possesses low impurity content (here, we do not include the occurrence of CaCO_3_—calcite, as a mineral within the composition of the raw, the starting material; CaCO_3_ exists at a temperature of 550 °C since the calcite decomposes thermally only at 700/750 °C) (Figure 4), compared with the traditional carbon-based reducers, such as the coal or coke. It is interesting to note that the purity of produced carbon helps to minimize contamination of the silicon product (Figure 4), which is especially important when producing high-purity silicon, for example, in the case of semiconductors or solar cells. Similar to the observations presented in the FTIR spectrum (Figure 3), TiO_2_ (the titanium dioxide) is present in the carbonized sample, which is characterized by the diffraction peak of anatase at 2*θ* = 57.2°, with (211) crystal plane [45], confirming the presence of catalyst material in CFR char carbon (Figure 4). Of course, the obvious presence of calcite (Figure 4) in CFR char carbon, both in the form of precipitated calcium carbonate (PCC) or ground calcium carbonate (GCC), used as the charged filler in the increasing of the strength properties of the filter paper [46], is in excellent agreement with the results regarding this identification in FTIR spectrum (see above—Figure 3).

Likewise, two smaller XRD peaks were observed at locations of 2*θ* = 18.9° and 2*θ* = 12.4°. The first mentioned XRD peak at 2*θ* = 18.9° is located within the amorphous region belonging to (10Ῑ) reflection of cellulose residue [47]. The second XRD peak at 2*θ* = 12.4° is attributed to the crystalline peak of cellulose derivative (CA) [48], which has not undergone complete degradation, taking into account the applied operating temperature. In addition, in the XRD spectrum, a strong and sharp peak was observed at the position of 2*θ* = 9.3° (Figure 4), and this diffraction peak shifts to a lower Bragg angle, corresponding to the (020) crystallographic plane of the polysaccharide entity [49,50], which is formed during the observed carbonization process, as a consequence of hydrolysis reactions at lower temperatures. The observed peak is evidence that carbon restructuring occurs during the thermal conversion of raw material, where the acyl moieties (‘functional groups’) can be incorporated into the layer, creating an extra space (which violates the hydrogen bonding) that leads to the looser polymer layer structure, but with increased layer thickness. This leads to a visible sharpness of the XRD peak (Figure 4), which increases the crystallinity [51] of the synthesized char carbon. However, it should be clearly noted that the sharp peak at 2*θ* = 9.3° could also originate from the presence of the crystalline structure of cellulose-II [52] as a consequence of the thermal response of the cellulose structure along the temperature change at a molecular level. So, the current diffraction peak may be an indicator that the part of the cellulose-I structure is transformed into the cellulose-II structure at elevated temperature (the structural alternation may occur at temperatures far below 550 °C) [53]. Consequently, this result implies the changing of the structural pattern of the cellulose crystals, and the latter is strongly related to the weakening of hydrogen bond strength. Finally, the diffraction peak positioned at a very low Bragg angle of 2*θ* = 6.3° can be clearly seen (Figure 4). This peak indicates a “liberated” carbon (C) with a prominent diffraction peak and corresponds to the (111) plane with a calculated *d*-spacing of ~1.40 nm (Figure 4), in harmony with Bragg’s law [54]. The latter may indicate that the produced CFR char carbon has a high-quality morphology of pores with similar pore replications!

### 3.3. GC-MS Analysis of CFR Char Carbon

**Figure 5:**
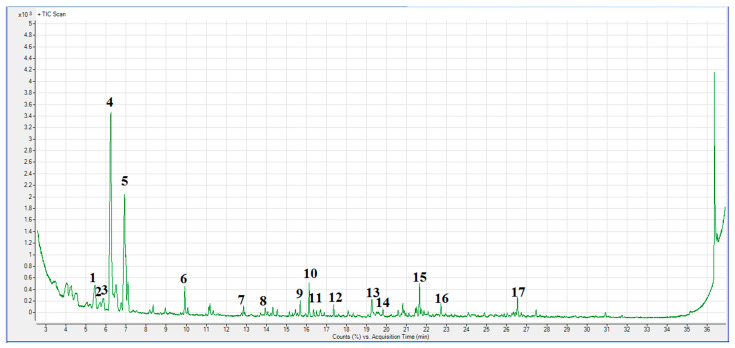
GC-MS chromatogram in the form of Counts (%) versus Acquisition Time (min) or Intensity (cps) (cps: counts per second) against Acquisition Time (min) for CFR char carbon (*T* = 550 °C; *t_res_* = 1 h).

GC-MS technique was used to determine the molecular composition of CFR-derived char carbon by carbonization at a not-so-high operating temperature (550 °C), as well as the influence of feeding material type on char pyrolysis fingerprint. GC-MS data may provide valuable information about the chemical composition and structure of the produced carbon, where differences in the above-mentioned concepts can have a significant effect on the role and fate of CFR char carbon in terms of its application. In this context, Figure 5 shows the GC-MS chromatogram of the obtained CFR char carbon (*T* = 550 °C; *t_res_* = 1 h).

Table 2 lists the typical carbolyzate components for CFR char carbon.

As a result of volatilization during pyrolysis, the manufactured solid by-product (in our case, synthesized CFR char carbon) is abundant with hydrocarbons family, especially most abundant of C_8_H_16_, C_9_H_18_, C_12_H_26_, and C_19_H_40_ compounds (Figure 5 and Table 2). Namely, the actual results may indicate that formed alkanes, especially three different n-alkanes (dodecane, tetradecane, and octadecane), can be used as phase change material (PCM) support [55]. So, the biocarbon produced from CFRs by the carbonization process at moderate temperature actually represents an introduced phase change material (PCM) (see the results in Table 2). In other words, through the applied thermal process, PCMs were infiltrated in the CFR char carbon network, forming char(*C*)/n-alkane composite material, which can have high latent heat storage capacity [55] (Table 2—the contribution of components such as octadecane, and then dodecane and tetradecane). The infiltration ratio of PCM in the char carbon can be large (over 50%), improving the char thermal stability and its chemical compatibility. All of this may have favorable morphological and structural properties (e.g., the quite large BET surface area and the mesopore structure) of CFR char carbon (see later results). Therefore, the results so far indicate that the resulting product could also be effectively used in thermal energy management systems (including energy storage and conversion applications).

Finally, it is interesting to highlight the emergence of organometallic ether compounds such as cyclohexyl(dimethoxy)methylsilane (Table 2) in the chemical composition of CFR char carbon since this compound may represent the char coupling agent. This means that its presence can improve char hydrophobicity and waterproof ability [56].

### 3.4. SEM-EDS: Morphology and Qualitative Elemental Analysis of CFR Char Carbon

The morphology of CFR char carbon was observed using SEM images at different magnifications to understand the effects of operating temperature and the residence time (under the intermediate carbonization conditions), while the EDS was employed for the qualitative elemental analysis of the produced char.

**Figure 6:**
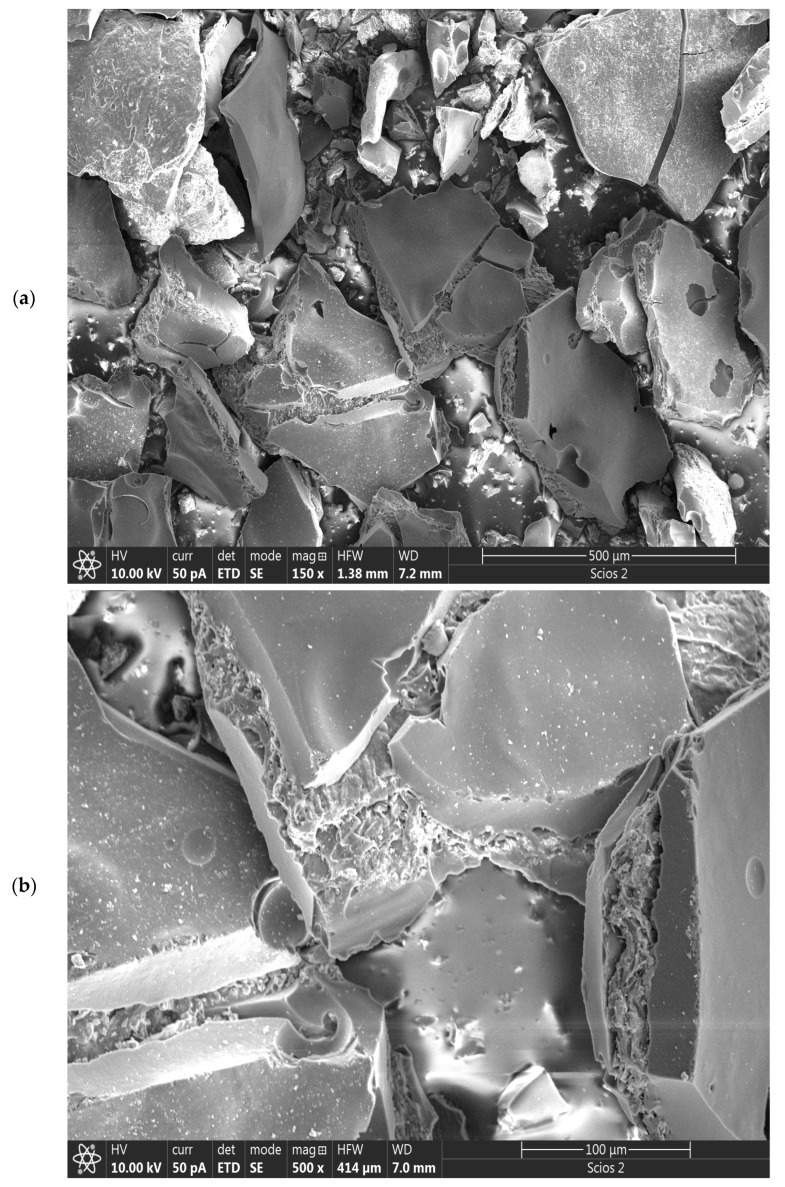

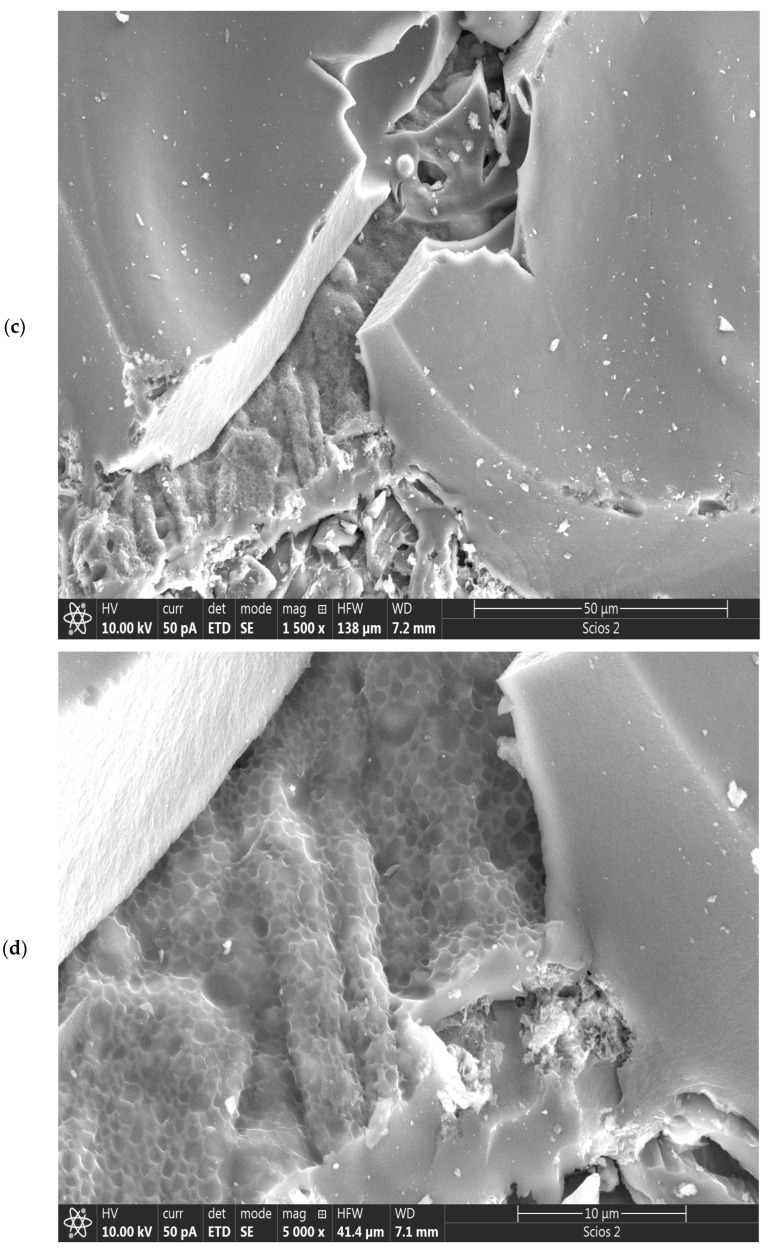

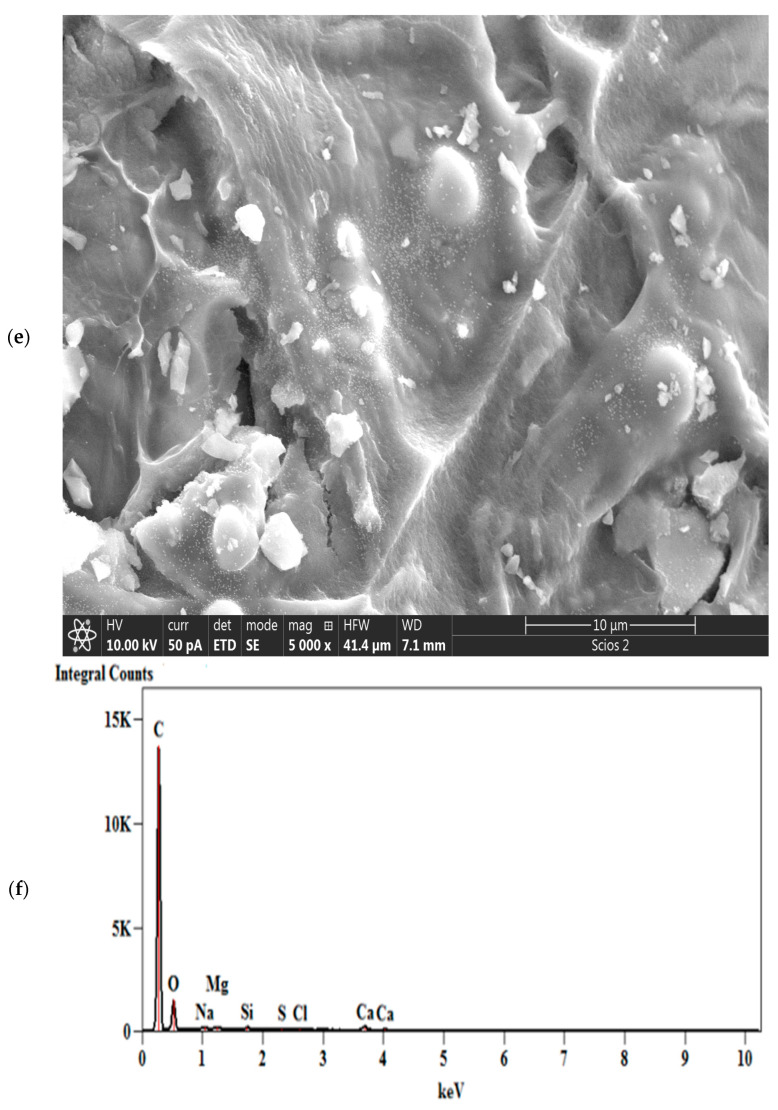
SEM images of CFR-derived char carbon at *T* = 550 °C and *t_res_* = 1 h, including different magnifications: (**a**) 150×, (**b**) 500×, (**c**) 1500×, (**d**) 5000×, and (**e**) 5000× (HWF: Horizontal field width; WD: Working distance). The EDS image of CFR-derived char carbon at *T* = 550 °C and *t_res_* = 1 h (**f**).

Figure 6a–e shows the SEM images of the CFR-derived char carbon by one-step carbonization process at* T* = 550 °C and *t_res_* = 1 h. The SEM image at the low magnification (150×) (Figure 6a) demonstrates the morphological structure of the carbonized sample that has not undergone full graphitization (some reduced degree of graphitization, but not minimized), where more irregular arrangements of carbon and the inorganic portions on the surface are formed, during its production. There are various “islands” with rougher and more uneven surfaces as well as smoother surfaces, with visible cracks in the form of holes, but also by an increased surface area and the porosity as larger and better-defined pores formed (Figure 6a). The effect of operating temperature, which is moderate (~550 °C), can be seen better in the SEM image at higher magnification (500×) (Figure 6b). On the right side of Figure 6b, the actual SEM image shows a split carbon block, in which there are remnants of the fibrous undecomposed cellulose structure and probably infiltrated in the pore structure, the long-chain hydrocarbons. They serve as a smokeless (clean and efficient) solid supporting medium, thus providing the CFR char with high compressive strength improvement, thermal stability, and mechanical performance. The same can be seen in the central part of Figure 6b, in a larger area. In this regard, it should be noted that two very weak vibrational peaks are observed at approximately ~2853 cm^−^^1^ and ~2922 cm^−^^1^ in the FTIR spectrum of CFR char carbon (they are not indicated in Figure 3), ascribed to the C–H stretching vibrations of methyl and methylene groups of n-alkanes [57]. Namely, in Section 3.1, it was said “*the absence of bands about 2900 cm^−1^–2800 cm^−1^ for the methylene groups, and overlapped bands from charred cellulose structure, with the inorganic components in the CFR char carbon spectrum, indicate a significant increase of condensed aromatic structure, and the loss of aliphatic functional groups due to…*” is, in fact, a correct statement, but the aforementioned bands, which are of extremely low intensity, actually originate from the existence of the “*pristine*” n-alkanes, which were confirmed by GC-MS analysis (see Figure 5 and Table 2). On the other hand, the assertion that “*…at lower wavenumbers, i.e., at 871 cm^−1^ up to 500 cm^−1^, are attributed to the deformation vibrations of C–H bonds present in aromatic rings*” (Section 3.1) is maintained, but in this wavenumber region, it may appear the absorption peaks which are characteristic for in-plane rocking vibration of the methylene repeating group (–(CH_2_)*_n_*–) [58]. Therefore, we can assume that n-alkanes are effectively ‘*encapsulated*’ into the pores of the CFR char.

However, special structures such as the reactive surface functional groups, microstructure, and pore characteristics enhance the comprehensive performances of fabricated char carbon. In the SEM image at much higher magnification (1500×) (Figure 6c), it can be observed that the produced CFR char carbon has a very well-developed pore structure, with dominant larger pore sizes (as average ~1–2.5 μm) (Figure 6c,d). The fine comb-like carbon/GO structure prevails (Figure 6c), which ensures the shaped stabilized material. Namely, this structure is interesting because it resembles a higher hierarchical order that corresponds more to “*ecdysis*” *C*-structure (››snake shedding skin‹‹ structure) (see Figure 6d—the central part, and to the lower left corner of the current SEM image). Both in Figure 6b and in Figure 6c,d with different magnifications, a large number of inorganic particles can be observed, mainly on the surface, from which they can be clearly distinguished calcium carbonate (CaCO_3_) sphere-shaped particles [59], as well as angular silicon (metallic) particles. Some of them create small aggregations. All of the above can be clearly seen in the high-magnification SEM image of 5000× (Figure 6e), where the accumulation of the minerals and silicon takes place on the rougher (more uneven) surface of the created carbon.

The results of the SEM analysis are directly confirmed by the EDS results, where Figure 6f presents the EDS spectrum of the elemental distribution in CFR char carbon. Their representation, expressed in the weight percentages and atom percentages, is shown in Table 3.

From the results listed in Table 3, the main elements can be clearly identified by their representation in the following order (weight in %): C (62.649%), O (28.660%), Ca (5.955%), and Si (0.969% ≈ 1%).

**Figure 7:**
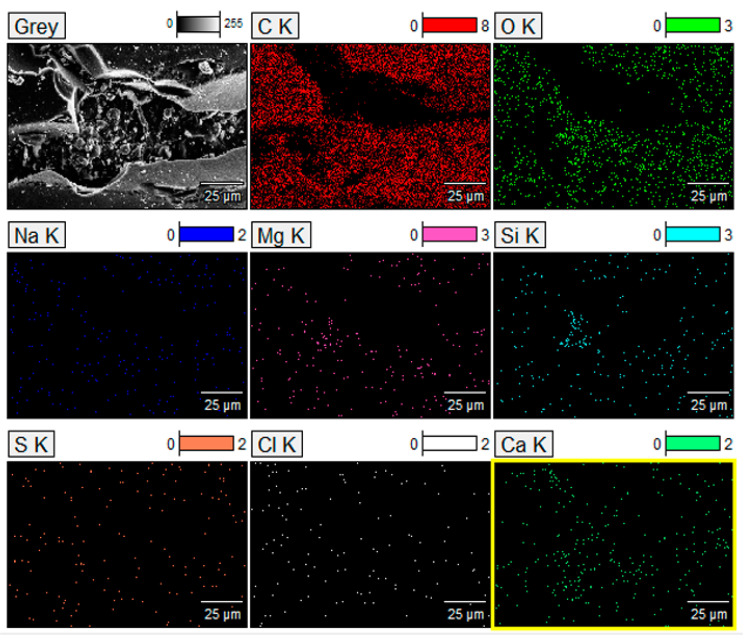
Mapping images of major element distribution (i.e., red shading for C, light green shading for O, blue shading for Na, pink shading for Mg, cyan shading for Si, orange shading for S, white shading for Cl, and blurred green shading for Ca) in the derived CFR char, characterized by the Field-emission scanning electron microscope (FESEM) combined with energy-dispersive X-ray spectroscopy (EDS) (the higher energy K line type was used).

The dominant element distribution characterized by the SEM combined with the EDS technique is shown in Figure 7. The elemental composition analysis was performed on the extracted rectangle shape of the SEM image portion of the sample in grey shading (Figure 7).

It can be observed from the results shown in Figure 7 that large concentrations of C and O are present and make a fully integrated carbon (amorphous)/GO network structure, where both were concentrated in a similar position. However, the participation of Ca and Si increases on the surface, thus leading to a decrease in the relative content of C distribution on the surface. Therefore, in some way, Ca and Si modify the produced CFR char, thus increasing its contents, and this is similar to the quantitative element analysis shown in Table 3. Carbon is the dominant component of the CFR char, followed by oxygen. As with previous confirmed results, a fairly large presence of Ca is evident, and to a somewhat lesser extent, silicon is also present, mainly at the surface of the char. However, the presence of the alkaline earth metal (Ca—through calcite) and metalloid (Si) link in the CFR char can reduce the pore size and specific surface area but significantly increase the average particle size of produced char, and this may lead to the “modifier” loading on the surface, or even entering into the pore structure (Figure 6 and Figure 7). Sodium (Na) and magnesium (Mg) are present in small quantities, while sulfur (S) and chlorine (Cl) (Table 3; far below 1%) are typical the char “impurities”, appearing in the form of fine particles [60] on the surface of the carbon (Figure 6 and Figure 7). However, these“impurities” can be trapped on the outer surfaces and in the resulting “channels” and “caves” (Figure 6e).

Finally, it should be noted that the calculated O/C ratio of CFR char carbon was 0.343, which is typical for char or charcoal (between 0.2 and. 0.6) (namely, during the thermal treatment, an increase in the temperature leads to O/C devaluation (compared with the starting raw material—see above), and falling to almost half the value (the formed char carbon)). This value (O/C ~ 0.343) indicates low hydrophilicity [61] and improved hydrophobicity (view previous results). Considering these facts, a “steady” increase in the operating temperature and a decline in the O/C ratio can be expected, which describes more C (carbon) stability, hydrophobicity, and aromaticity of the produced char. All of the above conditions are met by CFR char carbon synthesized at *T* = 550 °C and *t_res_* = 1 h (see Section 3.1,Section 3.2,Section 3.3 and Section 3.4).

### 3.5. XPS Analysis of CFR Char Carbon

**Figure 8:**
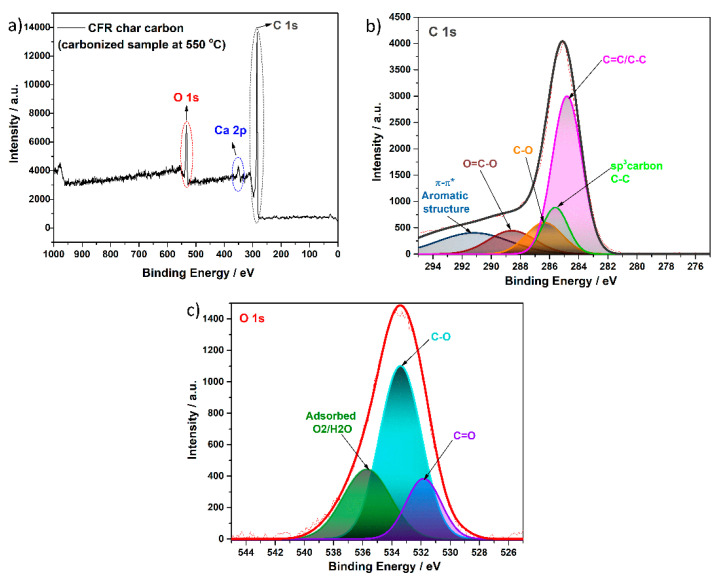
XPS spectra of the (**a**) surveys, (**b**) C 1s, and (**c**) O 1s of CFR char carbon (*T* = 550 °C, *t_res_* = 1 h).

The XPS analysis was also used for the structural characterization of the carbonized sample at 550 °C (CFR char carbon). The determined elemental compositions and the chemical state of surface elements are shown in Figure 8.

The survey spectra of the CFR char carbon sample (Figure 8a) show two peaks for carbon and oxygen atoms from the cellulose skeleton structure and signals for the inorganic component, such as Ca, in the form of CaCO_3_. This result is in full agreement with data obtained in the EDS analysis (see above), which shows that these elements have the highest percentage of contents in the sample. The deconvolution of the C 1s and O 1s (Figure 8b,c) spectra revealed the peaks in the 292 eV–280 eV and 545 eV–525 eV binding energy range, respectively. The C 1s (Figure 8b) peak was characterized by five deconvoluted peaks with binding energies of about 284.7 eV, 285.4, 286.3 eV, 288.6 eV, and 290.8 eV associated with C=C/C–C, sp^3^ carbon, C–O, O–C=O, and the π-π* system in the aromatic structure, respectively [62,63,64,65,66,67]. Additionally, the binding energy region, assigned to O 1s (Figure 8c), was found at 531.7 eV, 533.2 eV, and 535.5 eV, suggesting the presence of the C=O and C–O bonding, as well as chemisorbed oxygen or water in the sample, which is consistent with assignments in previous reports [62,65,68].

**Figure A1:**
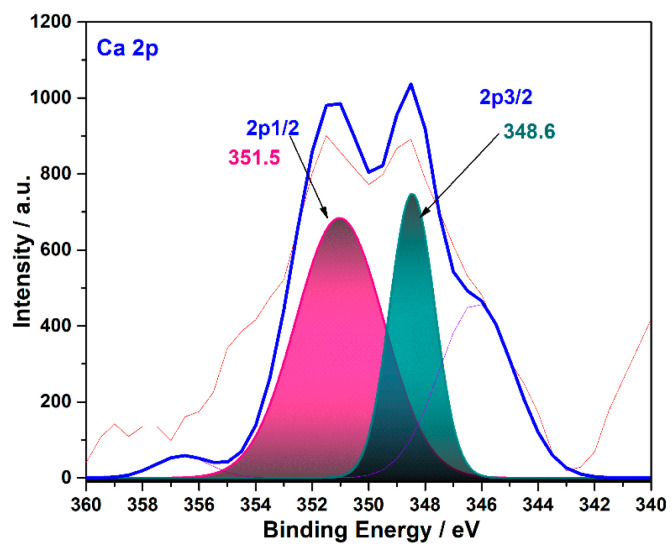
XPS spectra of the Ca 2p of CFR char carbon (*T* = 550 °C, *t_res_* = 1 h).

The band observed in the 360 eV–340 eV range originates from the Ca (2p) (Appendix A Figure A1). On the survey spectrum (Figure 8a), the small peak, observed at about 350 eV, is attributed to the 2p1/2 Ca orbit state, which is in accordance with previously cited literature data [24]. However, the presence of calcium (Ca) in both mentioned structures, in the form of CaCO_3_ present in the cigarette filter’ paper and the carbonized sample, is expected to be somewhat different in comparison to the pure commercial or the synthesized CaCO_3_. This result confirmed the presence of CaCO_3_, as already found using the XRD and FTIR techniques (see the results above).

### 3.6. Surface Area and Porosity Analysis of CFR Char Carbon

**Figure 9:**
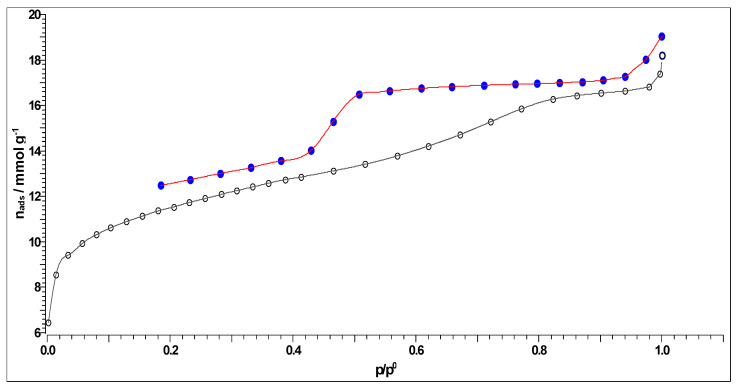
N_2_ adsorption (-○-)–desorption (-●-) isotherm of CFR char carbon, produced by the one-step carbonization process at *T* = 550 °C and *t_res_* = 1 h. Note 1: The observed hysteresis is quite broad, which is a sign of the pore system with a distinct polydispersity in size. Note 2: The adsorption (-○-) and the desorption (-●-) branches of the isotherm do not coincide, probably because of changes in the bulk density upon N_2_ sorption or irreversible adsorption of nitrogen. Additionally, the observed branch mismatching may occur because the condensation takes place within the mesopores of a given carbon material (there is a hysteresis pattern).

The surface area and the porosity of the char are the most important physicochemical characteristics evaluating the quantity and quality of active sites present, which increases many functional characteristics. Generally speaking, the macropores promote substance diffusion, the mesopores function as the mass transfer conduits, and the micropores serve as trapping and holding areas [69]. The nitrogen (N_2_) adsorption–desorption isotherm at 77 K for CFR char carbon is shown in Figure 9.

It can be observed from Figure 9 that the shown isotherm has an open loop (the apparent type H1 hysteresis), with the collaboration of the Type IV isotherms, according to the IUPAC manual [70], where it is characteristic for the mesoporous materials (for the CFR-derived carbon, the mesoporosity arises from the pyrolyzed CA, giving a preferential mesopore size), exhibiting capillary condensation processes within the pores. It should be noted that the open hysteresis loop observed in Figure 9 is typical for charcoal-type carbon. It seems to be typical for the appearance of very narrow slit pores or bottle-shaped pores. Namely, N_2_ adsorption in very narrow pores can be kinetically limited. So, the appearance of the obtained isotherm in Figure 9 corresponds to the adsorption on mesoporous solid material that proceeds via multilayer adsorption, followed by capillary condensation.

Table 4 lists the values of BET surface area, *S*_BET_ (m^2^/g), cumulative pore volume *V*_p(H-K)_ (cm^3^/g), median and maximum pore radius (*r_m_*_(H-K)_ and *r_max_*_(H-K)_) (nm) calculated by Horvath–Kawazoe (H-K) method (micropores analysis), micropore volume, *V*_micro_ (cm^3^/g), determined by *t*-Plot method (micropores analysis), cumulative pore volume, *V*_p(B_._J_._H_._)_ (cm^3^/g), median and maximum pore radius (*r_m_*_(B_._J_._H_._)_ and *r_max_*_(B_._J_._H_._)_) (nm) calculated by B.J.H. method (mesopores analysis), as well as the same quantities, determined by Cranston–Inkley (C-I) and Dollimore–Heal (D-H) methods (such as *V*_p(C-I)_, *r_m_*_(C-I)_, *r_max_*_(C-I)_, *V*_p(D-H)_, *r_m_*_(D-H)_ and *r_max_*_(D-H)_) (mesopores analysis), for the synthesized CFR char carbon.

**Figure 10:**
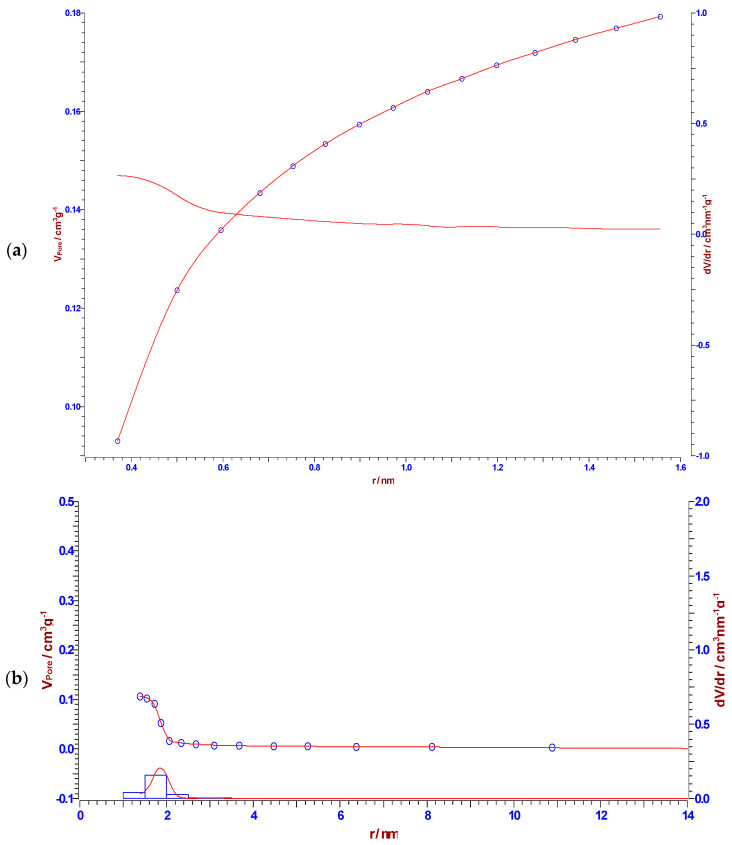

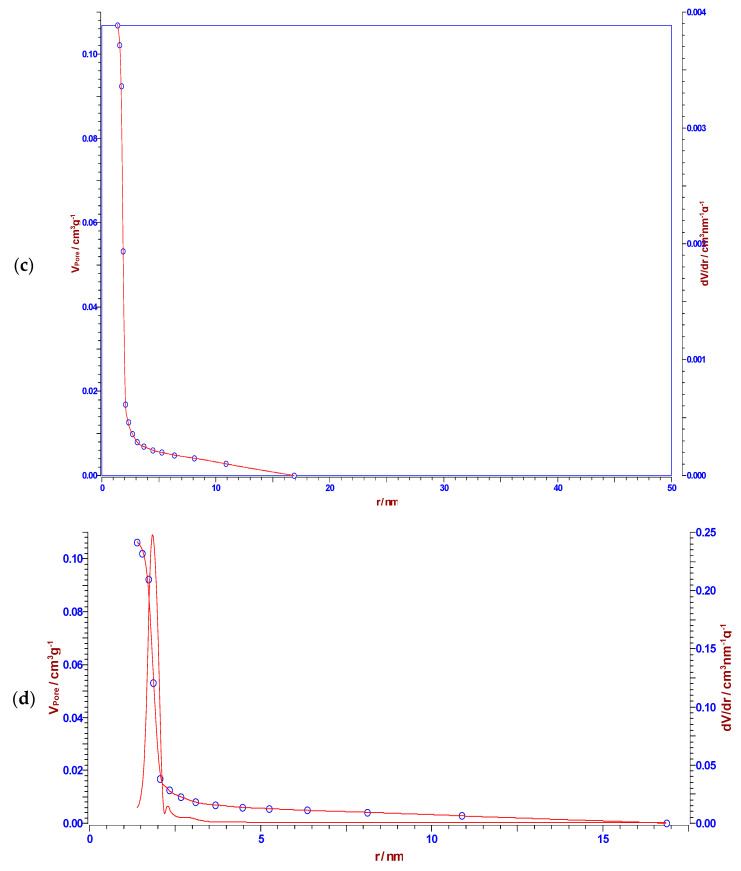
The pore size distributions were determined in accordance with the Horvath–Kawazoe (H–K) (**a**), B.J.H. (**b**), Cranston–Inkley (C–I) (**c**), and Dollimore–Heal (D–H) (**d**) methods, for CFR char carbon. A narrow size distribution was observed, which is characteristic of cellulose-based carbon.

It can be observed from the results presented in Table 4 that CFR char carbon is characterized by a high specific surface area of 320.93 m^2^/g, with a non-negligible fraction of micropore volume (0.0871 cm^3^/g). Namely, in Figure 9, at the lowest partial pressure range (up to *p*/*p*_o_ ~ 0.04), an almost linear slope occurs, which means that the small micropores are filled. The specified part of the isotherm corresponds to the behavior of the isotherm Type I(b), which is characteristic of materials with wider micropores, such as supermicropores (pore diameter between 0.7 nm and 2 nm) [71]. As the result of strong adsorbate-adsorbent interactions in the small micropores, an almost linear slope can be observed in the lowest partial pressure range (the limiting uptake is governed by the accessible micropore volume) [71]. On the other hand, the volume fraction of the mesopores is higher (0.1069 cm^3^/g), with a median pore radius of ~1.867 nm (Table 4). In addition, the representatively obtained pore size distributions (d*V*/d*r*), which were estimated by the Horvath–Kawazoe (H–K), the B.J.H., the Cranston–Inkley (C–I) and the Dollimore–Heal (D–H) methods, are shown in Figure 10a, Figure 10b, Figure 10c and Figure 10d, respectively. The B.J.H., Cranston–Inkley (C–I), and Dollimore–Heal (D–H) methods strictly assume that the capillary within a porous material is cylindrical. Considering that the desorption process has three major influences, such as the capillary condensate, the adsorbed layer, and the pore walls of varying pore sizes, the condensate can be found within the center of the pore and is retained there by the adsorbed layer, an ordered multilayer directly adsorbed to the pore wall. Each of the above-indicated methods aims to relate the total volume (Figure 10b–d) desorbed to the corresponding changes in the capillary diameter and adsorbed layer thickness. Based on the shapes of distributions, we may assume the presence of the slit pores and probably cylindrical pores (the pores less than the average mesopore size obtained from the N_2_ sorption can arise from the present microporosity and/or the existence of cylindrical pores, in the sorption analysis) in CFR char carbon. It should be indicated that pores of a smaller width may contain the same volume of a larger pore, which would inherently have a larger contribution to CFR char carbon because they would be more numerous.

However, in the current case, it should be emphasized that pore size from 2 nm to 50 nm corresponds to the definition of mesoporous materials, while CFR char carbon reaches a maximum pore radius of ~1.847 nm, which is very close to 2 nm (approaching an lower limit—frontier between microporous and mesoporous material), but very high specific surface area (much larger than that one reported in Table 4) can never be reached with a purely mesoporous material. Looking at the results of CFR char carbon porous properties, it is not fully capable of strong catalyst activities, controlled drug delivery, and gas adsorption applications. For these purposes, carbon materials that have pore sizes much larger than 2 nm are used.

Table 5 lists the contribution in the pore volume, *V*_p_ (cm^3^/g), and *V_pore_* (%), taking the most common range of pores in CFR char carbon, regarding the pore classes category.

The results in Table 5 clearly suggest that the majority of the volume is occupied by the pores with a diameter between 1.5 nm and 2 nm, and then followed by pores with diameters between 1 nm and 1.5 nm (*V_pore_* = 18.37%), then between 2 nm and 2.5 nm (*V_pore_* = 11.72%) as well as between 2.5 nm and 3 nm (*V_pore_* = 2.696%), and finally (without the last one in Table 5), between 3 nm and 3.5 nm (*V_pore_* = 1.077%). Apparently, the char produced belongs to the combined mesoporous and microporous carbon material classes.

The applied moderate carbonization conditions (the operating temperature of 550 °C and residence time of 1 h) probably lead to a more sophisticated evolution of the pores, with some potentially expanding while others contracting or narrowing. In any case, these conditions create a fine porosity without its intense collapsing, as would occur if the much higher temperatures (more than 700 °C) were applied. So, in a true sense, it can be expected that the pore volume and average pore size (the porosity) increase as the temperature rises up to ≈ 500 °C. After this temperature, pore coalescence may occur, while inorganic chemical species can further influence the regulation of the range of pore sizes created.

In this context, a comparison of the porosity of two carbon materials was performed, the one synthesized in the study under consideration and the one obtained from the cigarette butts (CBs) waste by the one-step high temperature (800 °C) carbonization process (Table 6), and the reported by the authors [14]. The comparison includes the carbonization (experimental) conditions, the porosity category of the material, the specific surface (BET) area (*S*_BET_), and the median pore radius.

Upon comparing the results from Table 6, it can be seen that the slower heating of considered feedstock and the moderate process conditions (especially with regard to the applied operating temperature) cause a significant increase in specific surface area at the expense of a decrease in pore sizes. The applied process yields much more aromatic carbons, twice as much, compared with the conditions used in obtaining the CAc800 (1 h) [14]. Consequently, in this study, manufactured carbon material represents a typical example of the production of char that contains both mesopores and micropores, a true combination of isotherm classes, i.e., Type I + IV (see Figure 9). So, the manufactured mesoporous char contains different amounts of micropores, which results in the larger adsorption at low adsorbate pressure, followed by an adsorption course that gives the shape of an isotherm [72], exactly as it is obtained in Figure 9. In conclusion, due to the complex hierarchical structure, CFR char carbon has a composite Type I/IV(a) isotherm (Figure 9) and includes a number of features typically associated with micro-/mesoporous materials. At low partial pressures, the adsorption isotherm exhibits a steep rise associated with micropore filling. The adsorption isotherm in the higher partial pressure range exhibits multilayer adsorption (Figure 9), followed by pore condensation, which is accompanied by apparent type H1 hysteresis. CFR char carbon also exhibits a sharp rise in adsorbed amount near saturation (Figure 9), associated with condensation in inter-particle voids. The presented results are in excellent agreement with the results given in Section 3.4.

### 3.7. Assessment of Possible Application of CFR Char Carbon

The physicochemical characterization of produced micro-mesoporous char carbon (via carbonization process from FRs) showed that this material could be used as electrode material in supercapacitors [73,74]. The advantage of the presented procedure is that a high-quality hierarchical micro/mesoporous carbon is synthesized without any external modification, i.e., its activation, either chemical or physical (active carbons), or even ultrasonication treatments.

Carbon materials play a fundamental role in electrochemical energy storage because of their appealing properties, including low cost, high availability, low environmental impact, surface functional groups, and high electrical conductivity, alongside thermal, mechanical, and chemical stability, among other factors. Currently, carbon materials can be considered the most extensively explored family in the field of supercapacitors and batteries, which are devices covering a wide range of applications demanding high power and high energy [75]. Porous carbon materials are the most commonly used materials for double-layer supercapacitors because of their high porosity, which provides a larger specific surface area. This characteristic enables greater ion accumulation on the material surface, resulting in higher energy storage capacity. The porosity of these materials facilitates effective ion transport between the electrolyte and electrode matrix, thereby reducing the resistance of supercapacitors and increasing their power.

The final properties of the char carbon obtained in this research strongly depend on the precursor used and the processing conditions employed. Based on established results in the actual study, some of the above-listed requirements for electrochemical energy storage applications of the produced char carbon are fulfilled but not completely. One of them is obtaining a very large specific surface area (ranging approximately between 2000 m^2^/g and 3000 m^2^/g), which would be reflected in the performance of produced carbon material, which may provide high capacitance. Therefore, such a large specific surface was not achieved by the strategy proposed here. Likewise, a specific surface modification was not carried out in this study to incorporate the various functional groups and enhance the electrochemical performance [76].

Therefore, it is necessary to expand this research in the future, including possible chemical and/or physical activation that would yield a greater uniformity of pores and specific surface area, introducing higher activation temperature/activation time. Namely, in addition to the specific surface area as an important parameter for the performance of electrochemical double-layer capacitors (EDLCs), other aspects of manufactured carbon material, such as pore size distribution, pore shape and structure, electrical conductivity, and surface functionality, can also influence its electrochemical performance to a great extent. Thus, additional efforts need to be made in the future, especially in the implementation of electrochemical tests, which is the authors’ primary goal for their next scientific paper.

## 4. Conclusions

This experimental research presents a methodology for obtaining a new type of carbon material from unused cigarette filter rods (CFRs) (marked as ‘CFR char carbon’) via a carbonization process under moderate conditions. It utilized a muffle tube furnace for the implementation of the process because it offers precise temperature control and streamlined operation to reach the desired heat level. The char was generated at the operating temperature of 550 °C and residence time of *t_res_* = 1 h, slowly applying heating (*β* = 3 °C/min). A complete physicochemical characterization was performed on the produced char carbon using various instrumental techniques, such as ATR-FTIR, XRD, GC-MS, SEM(FESEM)-EDS, XPS, and BET. As an important fact, the effect of the production method on the characteristics of the char carbon (which dictates its application) was highlighted. The X-ray powder diffraction (XRPD) and FTIR spectroscopy techniques have allowed for insight into the phase composition of the carbonized sample and to assess the structural characteristics of crystalline carbon material features (i.e., amorphous and crystalline phases within the studied material) as well as to identify and determine its surface functional groups. On the other hand, the morphological interpretation of produced char carbon was carried out using FESEM analysis (to obtain valuable information regarding the surface characteristics), while the elemental analysis of the prepared char was performed with the aid of EDS. Additional information about the molecular composition of CFR-derived char carbon was obtained using the GC-MS technique. XPS analysis of the prepared CFR char carbon provided information about its surface elemental composition, the chemical state, and the type of bonding present. Finally, aside from its chemical structure, the porosity-encompassing specific surface area and micro–mesopore structures are critical properties that significantly influence the performance of the produced char, regardless of the application. For this purpose, a detailed porosity analysis was performed, and pore distributions were derived using various methods. Based on all of the above studies, the following important conclusions were drawn:

- Moderate carbonization process conditions, predominantly a temperature of 550 °C and slow heating, lead to a higher yield of CFR char carbon, ranging between 17.9% and 18.6% (more than the yield of the char, derived from the biomass, ~12%).
- CFR char carbon is rich in oxygen functional groups, such as C=O, C–O, –C(=O)–CH_3_, C–O–C, C–OH, and O=C–O, with the existence of chemisorbed oxygen.
- The carbonized sample has the properties of typical porous carbon, where the graphitization stage is weakened. The sample is characterized by a well-formed aromatic structure. The presence of the graphene oxide (GO) pattern confirmed the expansion of interlayer spacing due to the introduction of oxygen functionalities. It was established that the diverse presence of oxygen-containing functional groups in CFR char carbon can have a significant effect on its structure and physicochemical properties, which could be used for practical applications.
- It was found that the obtained carbon material reaches the expansion of the interlayer spacing of 1.40 nm, which indicated that the product has a high-quality pore morphology with the possibility of pore replication.
- It was determined that the produced carbon has a fairly high content of mineral inorganic components, mostly alkaline earth metal such as calcium (Ca), that originates from the calcite (CaCO_3_—acts as the filler to increase the strength properties of the filter paper), then the silicon (Si), which is obtained by reducing SiO_2_ (where reducing agent represents CFR char carbon). The formed carbon (C) in the char product, due to the effects of elevated temperature, becomes a pertinent reactant in the process of silicon reduction to its pure form. In addition, the existence of TiO_2_ (titanium dioxide) in the carbonized sample was identified. TiO_2_ represents the stable phase (it was used as the filler and as a whitening agent in the cigarette filter) that plays a role as the catalyst material in CFR char carbon.
- Additional analysis of the chemical composition of CFR char carbon showed an abundance of long-chain hydrocarbons, and three different n-alkanes, such as dodecane, tetradecane, and octadecane, are of particular importance, acting as the phase change material (PCM) support. In the current study, it was assumed that due to the applied process conditions that form a given carbon material, PCMs infiltrated into the CFR char carbon network, forming a char(C)/n-alkane composite material, further strengthening it in terms of its stability (regarding thermal stability and chemical compatibility, together with high compressive strength improvement).
- The CFR-derived char carbon exhibits a very well-developed pore structure, where the higher hierarchical order morphology has been achieved. This structure yielded the main elements content in the following order: C (62.649%), O (28.660%), Ca (5.955%), and Si (0.969%). It was concluded that the presence of calcium (Ca—from the calcite) and metalloid (silicon—Si) particles in the char may reduce the pore size and specific surface area, which was established using porosity analyses.
- The obtained carbon material (CFR char carbon) showed a fairly large specific surface area (=320.93 m^2^/g), exhibiting a complex hierarchical structure which is characterized by the composite Type I/IV(a) isotherm attached to the micro-/mesoporous material.
- In comparison with similar starting (feeding) material for the synthesis of char carbon (cigarette butt (CB)-derived carbon was taken as an example), it was shown that the carbonization temperature as well as the heating rate and the type of the feedstock, have a key role for the production of carbon material with mesoporous properties (CBs-derived char) and carbon material with the complex hierarchical pores structure, including both, micropores and mesopores (CFR-derived char).
- It was concluded that additional electrochemical tests should be performed on the produced CFR char carbon and/or its activated successor to confirm the guidelines for its use in electrochemical energy storage applications.

## Figures and Tables

**Table 1:** The proximate (the content of moisture (M), volatile matter (VM), fixed carbon (FC), and ash) and the ultimate (the content of carbon—C, hydrogen—H, oxygen—O, and sulfur—S) analysis, for CFRs.

Proximate Analysis (%, *ar*. ^a^)		Ultimate Analysis (%, *daf* Basis ^b^) ^c^	
Moisture, M	4.26	C	49.43
Volatile matter, VM	87.89	H	5.80
Fixed carbon, FC	7.56	O	44.44
Ash	0.29	S	0.03

^a^ *ar*. As received (or as sampled) basis. ^b^ *daf*—Dry, ash-free basis. ^c^ Nitrogen (N) was not found.

**Table 2:** Typical carbolyzate components for CFR char carbon (*T* = 550 °C; *t_res_* = 1 h).

No.	Retention Time, RT (min)	Compound	Class of Organic Compounds	Main *m*/*z*
1	5.442	3-Ethyl-4-methylheptan-1-ol	Fatty alcohols	69, 84, 41
2	5.7	Cyclopentanone, 2-(1-methylpropyl)	Terpenoids	84, 55, 83
3	5.854	Hexane, 3-ethyl-2-methyl	Alkanes	43, 57, 84
4	6.484	1-Hexane, 4,5-dimethyl	Alkanes	43, 71, 41
5	6.924	2,4,4-trimethyl-1-hexene	Alkenes	43, 71, 70
6	9.94	Decane, 2,4,6-trimethyl	Alkanes	43, 57, 71
7	12.864	Silane, cyclohexyldimethoxymethyl	Organometallic	105, 75, 91
8	13.939	Decane	Alkanes	57, 43, 41
9	15.69	Undecane	Alkanes	57, 43, 41
10	16.131	Undecane, 2-methyl	Alkanes	43, 57, 41
11	16.137	Pentadecane	Alkanes	57, 43, 71
12	17.367	Dodecane	Alkanes	57, 43, 41
13	19.272	Tetradecane	Alkanes	57, 43, 41
14	19.822	Hexadecane	Alkanes	57, 43, 41
15	21.641	Nonadecane	Alkanes	57, 43, 41
16	22.717	Eicosane	Alkanes	57, 43, 41
17	26.534	Octadecane	Alkanes	57, 43, 41

**Table 3:** The elemental composition of CFR char carbon (*T* = 550 °C and *t_res_* = 1 h) determined using EDS analysis (Figure 6f).

Element	Intensity	Net Counts	Weight (%)	Atom (%)
C	511.012	79,406	62.649	71.932
O	53.665	8339	28.660	24.703
Na	1.287	200	0.244	0.147
Mg	2.671	415	0.454	0.257
Si	5.380	836	0.969	0.476
S	2.413	375	0.498	0.214
Cl	2.079	323	0.571	0.222
Ca	12.311	1913	5.955	2.049
Total			100.000	100.000

**Table 4:** Porous characteristics of CFR char carbon, produced at *T* = 550 °C and *t_res_* = 1 h.

Sample	*S*_BET_ (m^2^/g)	*V*_p(H-K)_ (cm^3^/g)	*r_m_*_(H-K)_ (nm)	*r_max_*_(H-K)_ (nm)	*V*_micro_ (cm^3^/g)	*V*_p(B.J.H.)_ (cm^3^/g)	*r_m_*_(B.J.H.)_ (nm)	*r_max_*_(B.J.H.)_ (nm)	*V*_p(C-I)_ (cm^3^/g)	*r_m_*_(C-I)_ (nm)	r_max__(C-I)_ (nm)	V_p(D-H)_ (nm)	*r_m_*_(D-H)_ (nm)	r_max__(D-H)_ (nm)
CFR char carbon (550 °C and 1 h)	320.93	0.1793	0.362	0.982	0.0871	0.1069	1.867	1.8468	0.1069	1.867	1.8421	0.1062	1.8679	1.8422

**Table 5:** The values of *V*_p_ and *V_pore_* for the most frequent occurrence of pore diameters in CFR char carbon, regarding pore classes category.

CFR Char Carbon		
Pore Classes [from (nm) to (nm)]	*V_p_* (cm^3^/g)	*V_pore_* (%) ^a^
1–1.5	0.0196	18.37
1.5–2	0.0792	74.1
2–2.5	0.0125	11.72
2.5–3	0.0029	2.696
3–3.5	0.0012	1.077
3.5–4	0.00069	0.649

**^a^** Mesopores’ surface area was 175.67 m^2^/g.

**Table 6:** Comparison of the porosity characteristics between CAc800 (1 h) (produced from CBs waste as starting raw material) sample [14] and CFR char carbon sample (this study).

Sample	Carbonization Conditions	Porosity Category of the Material	*S*_BET_ (m^2^/g)	Median Pore Radius (nm)	Reference
CAc800 (1 h)	Furnace, Heating rate = 4 °C/min, *T* = 800 °C, *t_res_* = 1 h	Mesoporous	55.65	3.17	[14]
CFR char carbon	Furnace, Heating rate = 3 °C/min, *T* = 550 °C, *t_res_* = 1 h	Microporous/ Mesoporous	320.93	1.867	This study

## Data Availability

The original contributions presented in this study are included in the article. Further inquiries can be directed to the corresponding author.

## Abbreviations

The following abbreviations are used in this manuscript:

CA	Cellulose acetate
CBs	Cigarette butts
FRs	Filter rods
CFRs	Cigarette filter rods
CFR char carbon	Cigarette filter rod char carbon
M	Moisture
VM	Volatile matter
FC	Fixed carbon
ATR-FTIR	Attenuated total reflectance—Fourier-transform infrared spectroscopy
XRD	X-ray diffraction
XRPD	X-ray powder diffraction
GC-MS	Gas Chromatography–Mass Spectrometry
SEM-EDS	Scanning electron microscopy and Energy-Dispersive X-ray spectroscopy
XPS	X-ray photoelectron spectroscopy
FESEM	Field-emission scanning electron microscope
EDS	Energy-dispersive X-ray spectroscopy
BSEI	The backscattered electron imaging
BET	Brunauer–Emmett–Teller
BJH	Barrett–Joyner–Halenda
CI	Cranston–Inkley
DH	Dollimore–Heal
HK	Horvath–Kawazoe
PCC	Precipitated calcium carbonate
GCC	Ground calcium carbonate
GO	Graphene oxide
RT	Retention time (min)
PCMs	Phase change materials
EDLCs	Electrochemical double-layer capacitors
